# Tetrathiomolybdate sensitizes ovarian cancer cells to anticancer drugs doxorubicin, fenretinide, 5-fluorouracil and mitomycin C

**DOI:** 10.1186/1471-2407-12-147

**Published:** 2012-04-13

**Authors:** Kyu Kwang Kim, Thilo S Lange, Rakesh K Singh, Laurent Brard, Richard G Moore

**Affiliations:** 1 Molecular Therapeutics Laboratory, Program in Women's Oncology, Departments of Obstetrics and Gynecology, Women and Infants Hospital, Alpert Medical School, Brown University, 101 Dudley Street, Providence, RI 02905, USA; 2 Department of Molecular Biology, Cell Biology, and Biochemistry, Brown University, Providence, RI 02912, USA; 3 Division of Gynecologic Oncology, Department of Obstetrics and Gynecology, Southern Illinois University School of Medicine, Springfield, IL 62794, USA

**Keywords:** Tetrathiomolybdate, Combination treatment, Doxorubicin, Mitomycin C, Fenretinide, 5-fluorouracil, ROS generation

## Abstract

**Background:**

Our recent study showed that tetrathiomolybdate (TM), a drug to treat copper overload disorders, can sensitize drug-resistant endometrial cancer cells to reactive oxygen species (ROS)-generating anticancer drug doxorubicin. To expand these findings in the present study we explore TM efficacy in combination with a spectrum of ROS-generating anticancer drugs including mitomycin C, fenretinide, 5-fluorouracil and doxorubicin in ovarian cancer cells as a model system.

**Methods:**

The effects of TM alone or in combination with doxorubicin, mitomycin C, fenretinide, or 5-fluorouracil were evaluated using a sulforhodamine B assay. Flow cytometry was used to detect the induction of apoptosis and ROS generation. Immunoblot analysis was carried out to investigate changes in signaling pathways.

**Results:**

TM potentiated doxorubicin-induced cytotoxicity and modulated key regulators of apoptosis (PARP, caspases, JNK and p38 MAPK) in SKOV-3 and A2780 ovarian cancer cell lines. These effects were linked to the increased production of ROS, as shown in SKOV-3 cells. ROS scavenging by ascorbic acid blocked the sensitization of cells by TM. TM also sensitized SKOV-3 to mitomycin C, fenretinide, and 5-fluorouracil. The increased cytotoxicity of these drugs in combination with TM was correlated with the activity of ROS, loss of a pro-survival factor (e.g. XIAP) and the appearance of a pro-apoptotic marker (e.g. PARP cleavage).

**Conclusions:**

Our data show that TM increases the efficacy of various anticancer drugs in ovarian cancer cells in a ROS-dependent manner.

## Background

Ovarian cancer is the second most common gynecological malignancy and the leading cause of gynecologic cancer deaths in the United States [1]. The initial treatment for women diagnosed with ovarian cancer includes cytoreductive surgery followed by platinum based adjuvant chemotherapy [2-4]. Although most women will have an initial response to primary treatment, most will eventually experience a recurrence with the development of chemoresistant disease. Improved chemotherapeutic agents and sensitizers for resistant tumors are needed.

In the present report we assess the effect of ammonium tetrathiomolybdate (TM) on the efficacy of a panel of common anticancer drugs in combination treatment against ovarian cancer cell lines *in vitro*. Due to the tetrahedral [MoS_4_]^2- ^anion, TM can function as a chelator and was first therapeutically used to treat copper toxicosis in Wilson's disease [5]. TM is known to decrease angiogenesis and cancer cell growth through the inhibition of cellular antioxidant copper zinc superoxide dismutase (SOD1) [6] and to elevate levels of cellular reactive oxygen species (ROS) [7,8]. Recent studies revealed that TM can also enhance the uptake and efficacy of cisplatin in human ovarian tumors [9]. Details to the mechanism of action by TM remain largely unknown. TM in tumor animal studies activated pro-apoptotic MAPK signaling, down regulated survival proteins, such as XIAP, and reduced cancer cell motility and invasiveness [10]. TM, in phase I trials in patients with a variety of metastatic cancers including breast, colon, lung, and prostate and in phase II trials in patients with advanced renal cancer and mesothelioma, was well tolerated and showed stabilization of the disease in a proportion of patients [11-13].

We recently reported that TM can sensitize drug-resistant endometrial cancer cell lines to doxorubicin [7]. The present study explores the ability of TM to potentiate the effect of doxorubicin and several other anticancer drugs including mitomycin C (MMC), fenretinide (4-HPR) and 5-fluorouracil (5-FU) in ovarian cancer cells. While these four drugs differ with respect to their application to treat ovarian cancer doxorubicin is used in recurrent disease, MMC showed activity in phase II trials, and 5-FU and 4-HPR showed positive responses in combination with platinum based drugs or as a single agent in phase II trials respectively [14-17]. These drugs were chosen for this study since they share one common target, the cellular oxidative defense system. Treatment with any of these drugs can lead to the elevation of oxidative stress promoting cell death in cancer cells [7,18-21]. Their efficacy may be improved if the oxidative balance in cancer cells is disrupted by agents that target cellular antioxidants such as TM. In the present study we describe the effect of TM combination treatment with doxorubicin, 4-HPR, 5-FU, and MMC on various cellular apoptotic markers and determine the correlation between ROS activity and cell death in ovarian cancer cells.

## Methods

### Cell lines, cell culture, and material

SKOV-3 (human ovarian adenocarcinoma) cells were purchased from ATCC (Manassas, VA) and A2780 (human ovarian adenocarcinoma) cells were supplied by Dr. Alexander Brodsky (Brown University, Providence, RI). Both cell lines were grown in Dulbecco's modified eagle's medium (DMEM) supplemented with 10% fetal calf serum or bovine calf serum and 1% penicillin/streptomycin; referred to as a complete medium. Cells were cultured at 37°C with 5% CO_2 _in a humidified incubator. Reagents were purchased as follows: Sulforhodamine B (SRB), doxorubicin, ammonium tetrathiomolybdate, phenylmethylsulfonyl fluoride, ascorbic acid, propidium iodide, rhodamine 123, 4-HPR and 5-FU (Sigma Aldrich, Madison, WI); MMC (Tocris Bioscience, Ellisville, MO); carboxy-H_2_DCFDA (Invitrogen, Carlsbad, CA); trichloroacetic acid and acetic acid (Fisher Scientific, Pittsburgh, PA); XIAP, JNK, p-JNK, p38, p-p38, cleaved PARP, cleaved caspase-3 and -7 antibodies were purchased from Cell Signaling Technologies (Danvers, MA, USA). GAPDH antibody was obtained from Santa Cruz Biotechnology (Santa Cruz, CA, USA).

### SRB cell viability assay

Cells were plated into a 96-well microtiter plate in complete medium and treated with TM (0, 30 μM) for 24 h, after which the cells were treated with either doxorubicin, MMC, 4-HPR, 5-FU of different concentrations and durations as indicated. For treatment with doxorubicin or MMC, 1 × 10^4 ^cells were seeded per well, for 4-HPR and 5-FU 7.5 × 10^3 ^cells were seeded per well. Cell viability, as represented by the amount of cell protein-bound SRB, was measured as previously described [22].

### Analysis of mitochondrial membrane potential (ΔΨ_m_)

SKOV-3 cells (1 × 10^5^) were seeded into 6-well plates and treated with TM (0, 30 μM) after which the cells were treated with doxorubicin (0, 5 μM) for another 24 h. The cells were incubated with rhodamine 123 (13 μM) for 30 min at 37°C prior to completion of the drug treatment. Rhodamine 123 is a cationic dye which localizes in the mitochondria of viable cells. The cells were harvested, resuspended in medium containing propidium iodide (7.5 μM) and analyzed by flow cytometry. Data was acquired on a BD FACSort flow cytometer using CellQuest software (BD Immunocytometry-Systems, San Jose, CA) and analyzed (ModFit LT software, Verity Software House, Inc., Topsham, ME). Ten thousand cells were analyzed for each sample.

### Western blot Analysis

SKOV-3 or A2780 cells were treated with TM and anticancer drugs (doxorubicin, MMC, 4-HPR or 5-FU) alone and in combination under the conditions indicated. Cells were lysed, protein concentration of the lysates quantified, proteins separated by NuPAGE system (Invitrogen, Carlsbad, CA), and immunoblotted as described previously [7].

### Detection of intracellular ROS

SKOV-3 cells (3 × 10^5^/well) were seeded into 6-well plates and treated with TM (0, 30 μM) for 24 h, after which the cells were additionally treated with doxorubicin (0, 10 μM) for another 5.5 h and the assay carried out as described previously [23]. After treatment, the cells were incubated with carboxy-H_2_DCFDA (25 μM) for 30 min at 37°C with 5% CO_2_. Carboxy-H_2_DCFDA is the acetylated form of a reduced fluorescein derivative that is cell-permeable and becomes fluorescent in the presence of cellular oxidants. Cells were harvested and resuspended in phosphate buffered saline buffer before being analyzed by flow cytometry.

## Results

### Tetrathiomolybdate increases the sensitivity of ovarian cancer cell lines to doxorubicin

**Figure 1:**
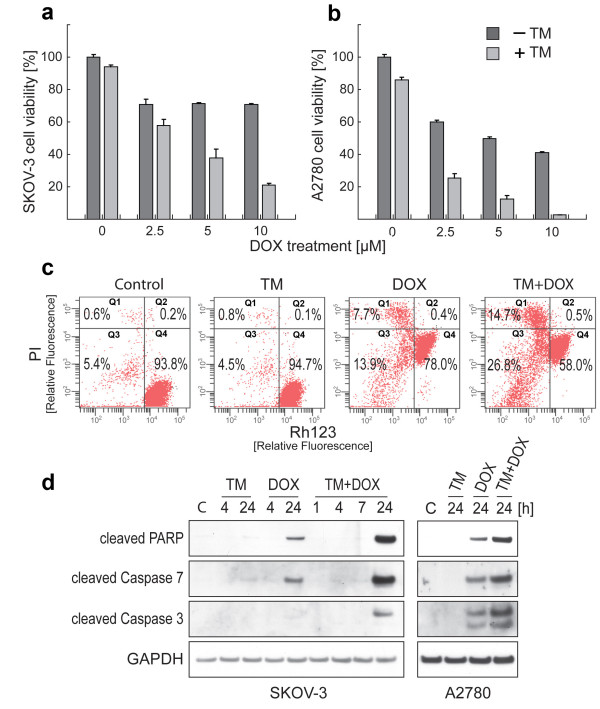
**Combinational treatment of ovarian cancer cell lines with TM and doxorubicin reduces cell viability via promotion of apoptotic signaling**. SKOV-3 (a) or A2780 (b) ovarian cancer cells were treated with TM (0, 30 μM for SKOV-3; 0, 7.5 μM for A2780) for 24 h, after which the cells were treated with doxorubicin (0, 2.5, 5, 10 μM) for another 24 h. Cell viability was evaluated as described in Methods. Data are expressed as the mean of the triplicate determinations (X ± SD) compared to untreated cells [100%]. (c) SKOV-3 cells were treated with TM (0, 30 μM) for 24 h, after which the cells were treated with doxorubicin (0, 5 μM) for another 24 h. The change in the mitochondrial membrane potential (ΔΨ_m_) was measured by flow cytometry. Intact cells = Q4, Loss of ΔΨ_m _= Q3, ruptured cell membrane = Q1 and Q2. (d) SKOV-3 cells (left panel) were treated with TM (0, 30 μM) for 24 h, after which the cells were treated with doxorubicin (0, 5 μM) for 1, 4, 7 or 24 h as indicated. The same procedure was employed to treat A2780 cells (right panel) with different concentrations of TM (0, 7.5 μM) and doxorubicin (0, 2.5 μM). Immunoblotting was carried out with primary antibodies against cleaved PARP, caspase-3, and -7. As an internal standard for equal loading blots were probed with an anti-GAPDH antibody.

To evaluate whether TM can sensitize ovarian cancer cells toward doxorubicin treatment, the SRB assay was carried out. Platinum resistant SKOV-3 ovarian cancer cells were treated with TM (0, 30 μM) for 24 h, after which the cells were treated with doxorubicin (0, 2.5, 5, 10 μM) for another 24 h. Cells treated solely with doxorubicin (following vehicle pre-treatment for 24 h) at all concentrations tested reduced cell proliferation by 29% as compared to untreated controls (Figure 1a). Cells treated solely with TM (30 μM) reduced cell viability by 5%. Pre-treatment with TM followed by treatment with doxorubicin revealed significant sensitization with viability inhibited by 42.1% (2.5 μM doxorubicin), 62.0% (5 μM) and 79.1% (10 μM). To verify TM-mediated sensitization of ovarian cancer cells to doxorubicin, platinum sensitive A2780 cells were treated as described above with the exception that TM was applied at the concentration of 7.5 μM. (Figure 1b). Treatment with TM alone resulted in 14% reduction of viability while doxorubicin alone exerted effects in a dose-dependent manner (41.7% viability reduction at 2.5 μM; 50% at 5 μM; 58.7% at 10 μM doxorubicin). The TM/doxorubicin combination revealed significant sensitization of A2780 cells with viability inhibited by 74.8% (2.5 μM doxorubicin), 87.5% (5 μM) or 97.1% (10 μM). Taken together, our results suggest that TM treatment sensitizes ovarian cancer cells to doxorubicin treatment *in vitro*.

### Doxorubicin-mediated apoptosis in ovarian cancer cells is enhanced by pre-treatment with TM

We carried out flow cytometry of drug treated cells (TM, doxorubicin, TM/doxorubicin combination) to measure a potential loss of mitochondrial membrane potential (ΔΨ_m_), an irreversible event during the process of apoptosis [24,25]. SKOV-3 cells were double-stained to detect cells with active mitochondria ΔΨ_m_, and to detect non-viable cells. As shown in Figure 1c, the cells treated with TM alone (30 μM, 48 h) only displayed minimal change in ΔΨ_m _and remained mostly viable (Q4; 94.7%). Similarly, the majority of cells treated with doxorubicin alone remained mostly viable (Q4; 78.0%) with 8.1% of cells (Q1 + Q2) being dead and an additional 13.9% displaying a disrupted ΔΨ_m _(Q3). In contrast, treatment with TM (30 μM, 24 h), followed by additional treatment with doxorubicin (5 μM, 24 h) increased the population of dead cells to 15.2% (Q1 + Q2) and also increased the population of cells with a disrupted ΔΨ_m _to 26.8% (Q3).

**Figure 2:**
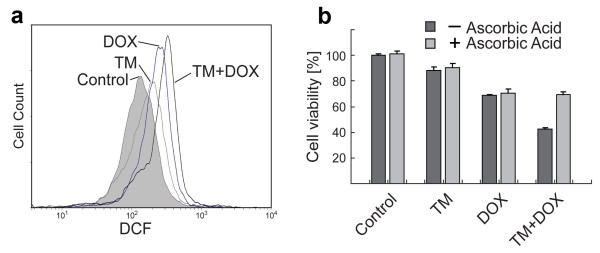
**Generation of intracellular ROS in ovarian cancer cells after individual or combinational treatment with TM and doxorubicin**. (a) SKOV-3 cells were treated with TM (0, 30 μM) for 24 h, after which the cells were treated with doxorubicin (0, 10 μM) for another 5.5 h. Generation of intracellular ROS was measured by flow cytometry (see Methods). Data are presented as relative fluorescence intensity (DCF). (b) SKOV-3 cells were treated with TM (0, 30 μM) for 24 h, after which the cells were treated with doxorubicin (0, 5 μM) for another 24 h in the presence or absence of radical scavenger ascorbic acid (750 μM) before the viability of cells was measured. Data are expressed as the mean of the triplicate determinations (X ± SD) compared to untreated cells [100%].

To define the cellular response of SKOV-3 cells upon TM and doxorubicin treatment we analyzed the activation of caspases characteristic for induction of apoptosis as well as the inactivation of PARP, a DNA repair factor, by immunoblotting. SKOV-3 cells were treated with TM (0, 30 μM) for 24 h, after which the cells were treated with doxorubicin (0, 5 μM) for 1, 4, 7 or 24 h as indicated (Figure 1d, left panel). PARP inactivation/cleavage and caspase-7 activation were observed when SKOV-3 cells were treated with doxorubicin alone for 24 h. TM/doxorubicin combination treatment, however, led to a strong detection of cleaved caspase-3 and to an increased level of activated caspase-7 and inactivated PARP when compared to doxorubicin treatment alone (Figure 2d, left panel). The same procedure was employed to treat A2780 cells (Figure 2d, right panel) with different concentrations of TM (7.5 μM) and doxorubicin (2.5 μM). Similarly to SKOV-3 cells, the most prominent cleavage of these apoptotic markers was found when A2780 cells were treated with TM/doxorubicin combination. Collectively, these results verify that TM sensitizes ovarian cancer cells to doxorubicin therapy and potentiates cell death via induction of apoptosis.

### An increased generation of intracellular ROS upon TM/doxorubicin combination treatment causes ovarian cancer cell death

One potential strategy suggested to treat cancer is to generate an excess amount of ROS in tumor tissue to induce cell death. We determined if SKOV-3 cell treatment with either TM or doxorubicin alone or in combination led to excess generation of ROS via flow cytometry. As shown in Figure 2a, ROS generation was slightly elevated over baseline after treatment with TM alone (shift in relative fluorescence intensity). Doxorubicin alone also caused a peak shift, which was further increased when the cells were first treated with TM followed by doxorubicin treatment.

To confirm that the generation of ROS by TM/doxorubicin combination treatment is the predominant mechanism of cytotoxic action we performed viability assays with SKOV-3 cells in the absence or presence of antioxidant ascorbic acid. As shown in Figure 2b the growth inhibitory effect of TM was not notably affected by ascorbic acid treatment (90.2% viability with ascorbic acid; 88.1% without). Similarly, scavenging of ROS did not reduce the cytotoxic action (70.4% viability with ascorbic acid; 68.9% without) by doxorubicin treatment. In contrast, in TM/doxorubicin combination treatment, ascorbic acid partially restored the viability (69.9% viability with ascorbic acid; 42.6% without) (Figure 2b).

### The potentiation of JNK and p38 activation by TM/doxorubicin combination treatment is ROS-dependant

**Figure 3:**
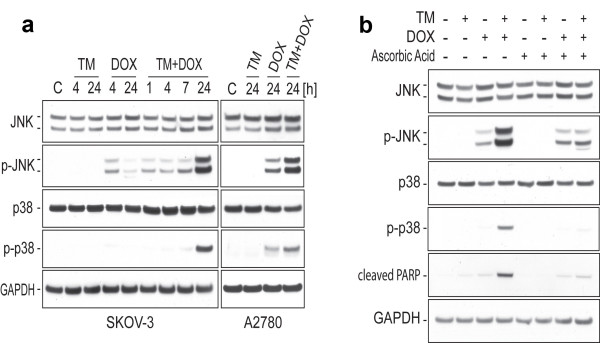
**Activation of pro-apoptotic JNK and p38 MAPK after TM/doxorubicin treatment with or without inhibition of ROS**. (a) SKOV-3 cells (left panel) were treated with TM (0, 30 μM) for 24 h, after which the cells were treated with doxorubicin (0, 5 μM) for 1, 4, 7 or 24 h as indicated. The same procedure was employed to treat A2780 cells (right panel) with different concentrations of TM (0, 7.5 μM) and doxorubicin (0, 2.5 μM). Immunoblotting was carried out with primary antibodies against native and activated/phosphorylated JNK or p38 MAPK. As an internal standard for equal loading blots were probed with an anti-GAPDH antibody. (b) SKOV-3 cells were treated as described in (a) in the presence or absence of radical scavenger ascorbic acid (750 μM). Immunoblotting was carried out with primary antibodies against JNK, p-JNK, p38, p-p38, cleaved PARP or GAPDH.

JNK and p38 are key regulators of stress-mediated apoptosis and activation of these MAPK takes a center stage in cellular response to treatment with TM/doxorubicin as shown recently for endometrial cancer cells *in vitro *[7]. By immunoblotting using primary antibodies against native and activated/phosphorylated JNK or p38 MAPK we analyzed the effect of TM/doxorubicin treatment on ovarian cancer cells. SKOV-3 cells were treated with TM (0, 30 μM) for 24 h, after which the cells were treated with doxorubicin (0, 5 μM) for various time periods as indicated (Figure 3a, left panel). The same procedure was employed to treat A2780 cells with different concentrations of TM (0, 7.5 μM) and doxorubicin (0, 2.5 μM) (Figure 3a, right panel). TM alone did not cause a change of JNK or p38 activation in SKOV-3 or A2780 cells. When cells were treated with doxorubicin for 24 h in combination with TM, a drastic increase in the activation of both MAPKs, compared to their activations in the cells treated by either TM or doxorubicin alone, was observed (Figure 3a).

To clarify whether an enhanced ROS production caused by the drugs alone or in combination potentiated the MAPK activations described above, we employed antioxidant ascorbic acid (750 μM) alongside to TM (30 μM) and doxorubicin (5 μM) treatment. As shown in Figure 3b, the potentiation of JNK and p38 activation upon TM/doxorubicin combination treatment was clearly attenuated by ascorbic acid, resulting in decreased kinase activation levels similar to those found in the cells treated with doxorubicin alone. Furthermore, the strong inactivation of PARP (an indicator for apoptotic processes) observed upon TM/doxorubicin combination treatment was blocked by ascorbic acid co-treatment and, thus, ROS dependent (Figure 3b). In summary, increased ROS generation caused by TM/doxorubicin combination potentiated cytotoxic effects (viability assay; Figure 2b) and pro-apoptotic signaling.

### TM induces sensitivity of ovarian cancer cells to various anticancer drugs via promotion of apoptotic signaling and ROS generation

**Figure 4:**
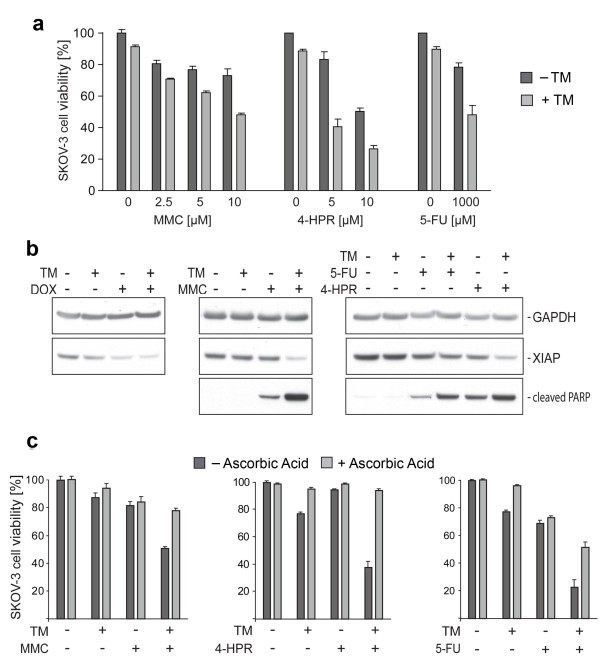
**TM increases ROS mediated cytotoxicity of various anticancer drugs in ovarian cancer cells**. (a) SKOV-3 cells were treated with TM (0, 30 μM) for 24 h, after which the cells were treated for another 24 h with MMC or for another 48 h with 4-HPR or 5-FU in the concentrations indicated. The cell viability was evaluated as described in Methods. Data are expressed as the mean of the triplicate determinations (X ± SD) compared to untreated cells [100%]. (b) SKOV-3 cells were treated with TM (0, 30 μM) for 24 h, after which the cells were treated for another 24 h with doxorubicin (0, 5 μM), MMC (0, 5 μM), 4-HPR (0, 10 μM) or 5-FU (0, 1000 μM). Immunoblotting was carried out with primary antibodies against cleaved PARP, pro-survival marker XIAP or GAPDH as internal control for equal loading. (c) SKOV-3 cells were treated with TM (0, 30 μM) for 24 h, after which the cells were treated for another 24 h with MMC (0, 5 μM) or for another 48 h with 4-HPR (0, 5 μM) or 5-FU (0, 1000 μM) in the presence or absence of radical scavenger ascorbic acid (750 μM).

A multitude of commonly used anticancer drugs display mechanistic features similar to those ascribed to doxorubicin when used to treat cells *in vitro*, such as the elevation of ROS or MAPK activation. We investigated if TM potentially can sensitize cancer cells to a panel of drugs including MMC, 4-HPR, and 5-FU. SKOV-3 cells were treated with TM (0, 30 μM) for 24 h, after which the cells treated for another 24 h with MMC or another 48 h with 4-HPR or 5-FU (Figure 4a). Cells treated solely with MMC (2.5, 5, 10 μM), similarly to doxorubicin, did not show a clear dose response (Figure 1a) and reduced cell viability by only 27% as compared to untreated controls. 4-HPR exerted dose-dependent cytotoxicity on SKOV-3 cells (viability of 83.1% at 5 μM; 50.4% at 10 μM). 5-FU displayed partial effects (viability of 78.3% at 1 mM). As seen for doxorubicin, treatment with TM potentiated the cytotoxic effect of all three drugs tested. Under these conditions the viability was reduced to 48.2% (TM + 10 μM MMC), 40.3% (TM + 5 μM 4-HPR) and 48.2% (TM + 1 mM 5-FU), respectively (Figure 4a). In summary, depending on the drug and concentration used, TM increased the cytotoxicity between 1.6 and 2.9 fold after collecting the effect of TM alone (Table 1). Our experiments suggest that TM treatment improves the response of ovarian cancer cells to doxorubicin, MMC, 4-HPR, and 5-FU treatment *in vitro*.

**Table 1:** Effect of drug combinations on SKOV-3 cell viability

Cell Viability [% of Untreated Control]	DOX [10 μM]	MMC [10 μM]	4-HPR [5 μM]	5-FU [1 mM]
No TM	70.7%	73.0%	83.1%	78.3%

With TM, 30 μM	20.9%	48.2%	40.3%	48.2%

Cytotoxicity Increase	2.5 fold	1.6 fold	2.9 fold	1.9 fold

To confirm that the potentiation of the cytotoxicity of these drugs by TM treatment is accompanied by suppressed anti-apoptotic events we analyzed the inactivation/cleavage of PARP, a DNA repair factor, and expression of the X-linked inhibitor of apoptosis (XIAP), by immunoblotting. SKOV-3 cells were treated with TM (0, 30 μM) for 24 h, after which the cells were treated with doxorubicin (0, 5 μM), MMC (0, 5 μM), 4-HPR (0, 10 μM) or 5-FU (0, 1000 μM) for another 24 h. As shown, PARP inactivation/cleavage over background levels (untreated controls) was observed for individual treatment with MMC, 4-HPR, and 5-FU (Figure 4b) as shown for doxorubicin (Figure 1d) but not for TM treatment. Combination treatment of SKOV-3 cells with TM potentiated the effects of all drugs tested on PARP. PARP was partially inactivated/cleaved after TM/5-FU treatment and drastically inactivated after TM/MMC, TM/4-HPR (Figure 4b) and TM/doxorubicin (Figure 1d) treatment. XIAP expression was reduced after TM/5-FU and barely detectable after TM/doxorubicin, TM/MMC and TM/4-HPR combination treatment (Figure 4b). In summary, TM sensitized ovarian cancer cells to treatment with these four anticancer drugs and potentiated cell death via induction of apoptosis.

We showed that the generation of ROS by TM/doxorubicin combination treatment is a key mechanism of cytotoxic action (Figure 2) in ovarian cancer cells. To assess if ascorbic acid can similarly quench the effects of 5-FU, MMC and 4-HPR when combined with TM, we performed viability assays using SKOV-3 cells. Cells were treated with TM (0, 30 μM) for 24 h, after which the cells were treated with MMC (0, 5 μM) for another 24 h or 4-HPR (0, 5 μM) or 5-FU (0, 1000 μM) for another 48 h. As shown in Figure 4c scavenging of ROS by ascorbic acid did not significantly reduce the partial cytotoxic effects of any single drug. In contrast, upon TM/MMC combination treatment, ascorbic acid restored cell viability effectively from 51.0 to 78.1%. Upon TM/4-HPR treatment, ascorbic acid restored cell viability dramatically from 37.6 to 94.1%. Upon TM/5-FU treatment, ascorbic acid restored the viability effectively from 22.7 to 51.3% (Figure 4c).

## Discussion

The present report reveals that combination treatment of ovarian cancer cells *in vitro *with TM improved the efficacy of a panel of anticancer drugs, namely doxorubicin, MMC, 4-HPR and 5-FU. We recently reported that TM sensitized drug-resistant endometrial cancer cell lines to doxorubicin [7] similar to ovarian cancer cells. Doxorubicin is among the most widely used agents to treat tumors, including lymphomas, leukemias, lung, breast, and ovarian cancers [16]. Doxorubicin is classified as a topoisomerase II inhibitor but its cytotoxic mechanisms remain unclear. The interaction of doxorubicin with cellular iron and its quinone moiety with oxo-reductive enzymes leads to the formation of radicals and excessive generation of ROS promoting cancer cell death [7,21,26,27]. It has been proposed that the pharmacological suppression of cellular antioxidants or elevation of ROS levels can sensitize tumor cells to doxorubicin [28,29] as shown here by combination treatment with TM. Such a treatment modality would provide a benefit to cancer patients because the use of doxorubicin and other anticancer agents is often challenged by concentration-dependent toxic effects [30]. Interestingly, independent of improved anti-tumor efficacy, TM treatment was shown in mice to protect against doxorubicin-induced cardiac toxicity [31]. Cardiotoxicity is the major limiting factor in the clinical use of doxorubicin implicating that combination therapy with TM may also provide clinical benefits secondary to the mitigation of doxorubicin toxicity during chemotherapy. Similar to doxorubicin, MMC contains a quinone moiety and leads to radical formation *in vitro *[19]. In addition, as for doxorubicin, the efficacy of MMC in ovarian cancer cells was improved and was linked to an increased ROS activity when the cells were treated in combination with TM as shown in the present report. MMC was originally isolated as an antibiotic from Streptomyces and is a potent DNA cross-linker classifying it as a potential agent to treat a broad range of solid tumors [32]. Phase II trials of low-dose MMC in patients with refractory ovarian cancer revealed higher survival rates and no significant toxicity [14]. MMC has also successfully been used alongside other drugs such as irinotecan (topoisomerase inhibitor) to treat refractory ovarian cancer [33]. Because TM was well tolerated in patients with Wilson disease and in phase I and II trials to treat patients with a variety of different tumors [11-13,34], and based on our findings we suggest to establish trials to treat resistant and/or refractory ovarian cancer by combination therapy with TM and doxorubicin or alternatively with MMC.

TM in ovarian cancer cells also potentiated the effect of the pyrimidine analog 5-FU which acts as a thymidylate synthase inhibitor, thereby strongly affecting rapidly dividing cancerous cells [35]. It has been shown previously that 5-FU can improve the efficacy of platinum based drugs in combination for the treatment of ovarian cancer [15,36]. 5-FU in these studies was well tolerated and can be used at relatively high concentrations without toxic side effects. The effects of anticancer drugs that are known to target DNA integrity or synthesis are often correlated to the induction of pro-apoptotic signaling and the generation of oxidative stress [7,20]. In the present report, the mild cytotoxicity displayed by 5-FU as a single agent was ROS independent while TM/5-FU combination treatment led to a significant ROS based reduction of ovarian cancer cell viability suggesting that expanded in vitro studies are needed to establish the parameters for the use of these two drugs in animal tumor models.

Similarly, our study suggests that the combination treatment with 4-HPR and TM can be considered to treat ovarian or other cancers. 4-HPR is a synthetic retinoid with antitumor activity and is known to cause cellular metabolic perturbations and induction of apoptosis in ovarian and other cancer cells via ROS-dependent mechanisms involving ER stress and MAPK (e.g. JNK) activation [18,37]. In recent phase II trials, patients with recurrent ovarian and primary peritoneal carcinoma received 4-HPR. It was well tolerated and displayed clinical responses [17]. Based on our findings, ovarian tumors may be sensitized to the treatment with 4-HPR by combination treatment with TM. Because both drugs were well tolerated by patients in previous studies, we suggest exploring the efficacy of TM/4-HPR in animal tumor models and ultimately in clinical applications.

As shown in the present report, TM increases the efficacy of doxorubicin, MMC, 4-HPR and 5-FU in ovarian cancer cells. Accordingly, lower concentrations of these drugs are needed to cause significant cytotoxicity. A similar effect during *in vivo *treatments could potentially reduce administered doses and toxic side effects of these or other anticancer drugs when combined with TM. The drug effects of doxorubicin, MMC, 4-HPR or 5-FU are potentiated when TM is co-applied to the cells unless an antioxidant is added; proving that increased ROS generation is responsible for drug action when used in combination. ROS are byproducts of normal cellular metabolism and tightly regulated in balance with cellular antioxidants. Cancer cells, through mitochondria dysfunction and increased metabolism, generate a relatively high level of ROS [38,39]. Further elevation of cellular ROS beyond a toxic threshold, as shown here after combinational treatment with TM, is an attractive strategy to selectively target tumor cells during chemotherapy [40,41]. These phenomena are not unique to TM combination treatment because ROS generation is a key mechanism of apoptosis for a variety of common chemotherapeutic drugs such as daunorubicin, cyclophosphamide or cisplatin [42-44]. Potentially, combination treatment of TM with doxorubicin, MMC, 4-HPR, or 5-FU may exert additional synergistic effects when combined with other agents thought to modulate the antioxidant functions of cancer cells such as 2-methoxyestradiol (SOD inhibitor) or drugs leading to glutathione depletion such as buthionine-sulfoximine. This approach might, in particular, be applied to treat MDR tumors because, for example, SOD1 is a therapeutic target of TM [6] and inhibition of SOD1 was shown to restore the cisplatin-sensitivity in resistant ovarian cancer cells [45]. It has been reported that chemotherapy-induced generation of ROS is often correlated with activation of the JNK and p38 MAPK signaling pathways [46,47] which play a crucial role in the response of ovarian cancer cells to common anticancer agents such as cisplatin [48]. Even though a detailed analysis of the modulation of these signaling pathways is beyond the scope of the present report we conducted a limited study to the effect of one drug combination (TM/doxorubicin) on the activation of these apoptotic markers in ovarian cancer cells. Combination treatment led to a dramatic increase of JNK and p38 activation which could be blocked by ROS scavenging and, thus, was directly correlated to the increase of ROS levels. Accordingly, apart from the destabilization of the cellular oxidative defense system, the subsequent potentiation of stress kinase signals also plays a vital role in the improved efficacy by TM combination therapy.

## Conclusions

The present study reveals that TM increases the efficacy of various anticancer drugs in multi-drug resistant ovarian cancer cells in a ROS-dependent manner. In addition, to the study of medical applications of TM, as discussed in the previous sections, we suggest to expand the analysis of biochemical and mechanistic effects of TM combination therapy *in vitro *and in animal tumor models. These may include the use of redox-modulating agents, cell lines derived from refractory ovarian or other cancers, and a broader panel of cancer drugs. Ultimately, the use of TM or TM-derivatives in combination therapy may improve current cancer treatment options.

## Competing interests

The authors declare that they have no competing interests.

## Authors' contributions

Coordinated the study (KK, TL, RS, RM, LB). Carried out the experiments (KK, RS). Analyzed the data (KK, TL). Wrote the manuscript (KK, TL, RS, RM, LB). All authors read and approved the final manuscript.

## Pre-publication history

The pre-publication history for this paper can be accessed here:

http://www.biomedcentral.com/1471-2407/12/147/prepub
